# The clinical investigator-subject relationship: a contextual approach

**DOI:** 10.1186/1747-5341-4-16

**Published:** 2009-12-03

**Authors:** David B Resnik

**Affiliations:** 1 National Institute of Environmental Health Sciences, National Institutes of Health, Box 12233, Mail Drop CU03, Research Triangle Park, NC, 27709, USA

## Abstract

**Background:**

The nature of the relationship between a clinical investigator and a research subject has generated considerable debate because the investigator occupies two distinct roles: clinician and scientist. As a clinician, the investigator has duties to provide the patient with optimal care and undivided loyalty. As a scientist, the investigator has duties to follow the rules, procedures and methods described in the protocol.

**Results and conclusion:**

In this article, I present a contextual approach to the investigator-subject relationship. The extent of the investigator's duty to provide the patient/subject with clinical care can vary from one situation to the next, as a function of several factors, including: the research design, benefits and risks of the research; the subject's reasonable expectations, motivations, and vulnerabilities; the investigator's ability to benefit the subject; and the investigator's prior relationship with the subject. These and other factors need to be considered when determining the clinical investigator's obligations to provide clinical care to human research subjects. In some research contexts, the investigator has extensive clinical obligations to the patient/subject; in others, the investigator has minimal ones.

## Background

What is the nature of the relationship between a clinical investigator^a ^and a human research subject? This topic has been a central concern in many controversies related to clinical research, including the use of placebo control groups, randomization, stopping clinical trials early, post-trial access to medications, informing subjects about incidental findings, provision of ancillary care, medication wash-out periods, and many others [1-21]. It has also taken center stage in some prominent legal cases, such as *Moore v. Regents of the University of California *[22], *Grimes v. Kennedy Krieger Institute, Inc *[23] and *Greenberg v. Miami Children's Hospital Research Institute *[24].

The nature of the relationship between a clinical investigator and a research subject has generated considerable debate because the investigator occupies two distinct roles: clinician and scientist [10]. As a clinician, the investigator has duties to provide the patient with optimal care and undivided loyalty. As a scientist, the investigator has duties to follow the rules, procedures, and methods described in the protocol. These distinct obligations may sometimes conflict when investigators are conducting clinical research.

For several decades, the fiduciary approach has been an influential view of the clinical investigator-subject relationship. According to this view, if an investigator's scientific duties conflict with obligations to provide the patient with optimal care, obligations to the patient/subject should prevail, because the investigator, as a fiduciary, should not place other interests above those of the patient [2,4,25,26].

Miller^b ^and Brody [6,7,16] and several other writers have criticized the fiduciary view [9,13]. While critics agree that investigators are not obligated to provide their patients/subjects with optimal care, they disagree about how much care investigators owe their patients/subjects. At one extreme, Miller and Brody [6,7,16] argue that investigators have minimal obligations to provide medical therapy to patients/subjects beyond what is required by the protocol or to protect patients from harm; at the other extreme, Richardson and Belsky [9] argue that investigators can have extensive obligations to provided beneficial therapy to patients/subjects.

In this article, I will present a contextual approach to the investigator-subject relationship. I will argue that the extent of the investigator's obligations to provide clinical care to the patient/subject can vary from one situation to the next. In some research contexts, the investigator has an obligation to provide the patient/subject with optimal care; in others, the investigator has only an obligation to provide the patient/subject with the care available under the protocol. There are also variations between these two extremes.

### Preliminary remarks on ethics and the law

The relationship between a clinical investigator and a research subject has ethical and legal dimensions [27]. While this paper will focus on ethical aspects of the investigator-subject relationship, the conclusions drawn herein could have ramifications for the law, because ethical arguments, distinctions, and considerations often influence legislation, administrative rule-making, and legal decisions [28,29]. For example, growing concerns about ethical abuses in research with human subjects, such as the infamous Tuskegee Syphilis Study, led to the passage of the National Research Act [30], which authorized federal agencies to codify regulations for the protection of human research subjects, and led to the appointment of a national commission to examine the ethics of research with human subjects. This commission drafted the *Belmont Report*, which provided a conceptual foundation for major revisions of the federal research regulations in 1981 [31]. Ethical considerations have also played an important role in legal cases concerning research with human subjects [13,32]. In *In re Cincinnati Radiation Litigation*, the court referred to the importance of the Nuremberg Code as an ethical standard for research, especially the code's emphasis on informed consent [33]. In *Grimes v. Kennedy Krieger Institute, Inc*., the court also mentioned the significance of the Nuremberg Code as a guide for research with human subjects and referred to it as part of the international common law [23].

### Historical perspective

To better understand the fiduciary view of the investigator-subject relationship, it will be useful place it in an historical context. Two distinct ideas relating to human research ethics came to fruition in the 1970s. The first idea emerged as a response to various abuses of human subjects, such as the Nazi experiments on concentration camp prisoners, the Tuskegee Syphilis Study, the Willowbrook Hepatitis Experiments, and other infamous cases [34,35]. As a reaction to these abuses, scholars and scientists argued that the rights, welfare, and dignity of research subjects must be vigorously protected, and policymakers enacted laws and regulations that reflected this consensus. The emerging *zeitgeist *emphasized the importance of protecting the rights and welfare of the patient/subject and was deeply skeptical of utilitarian perspectives on research [36-39]. A version of the Helsinki Declaration adopted in 1975 gave a clear statement of this viewpoint: "In research on man, the interest of science and society should never take precedence over considerations related to the well-being of the subject" [40]. The most recent version of the Declaration contains a similar statement [41].

The second idea emerged in response to growing ethical and philosophical tensions between medical research and practice. In 1979, the authors of the *Belmont Report *drew a distinction between research and practice in order to decide which activities needed to be reviewed for the protection of human subjects [42]. Clinical research activities were thought to require a layer of review over and above the professional peer review that occurs in medicine [43]. The authors also recognized that research and practice often take place concurrently and that sometimes innovative therapies are described as "experimental."

The *Belmont Report *defined research as an activity "designed to test an hypothesis, permit conclusions to be drawn, and thereby to develop or contribute to generalizable knowledge," and defined practice as an activity "designed solely to enhance the well-being of an individual patient or client" [[42] at p 5]. This definition of research has substantially influenced the development of law and policy around the world. When research and practice occur concurrently, as in a clinical trial, the activity should be reviewed as research, to ensure that human subjects receive adequate protections. Innovative therapies need not be classified as research, according to the *Report*, so long as they are designed solely to benefit the patient.

### Equipoise and the fiduciary approach

These two influential ideas collided when scholars became concerned that, while clinical research and clinical practice are conceptually distinct activities, the rights and welfare of patients participating in clinical are sometimes compromised in randomized clinical trials (RCTs). In placebo-controlled RCTs subjects are randomly assigned to receive the experimental treatment or a placebo. Subjects in the placebo group receive an inert, medically ineffective treatment for their condition, whereas subjects in the experimental group receive a therapy thought to be medically effective. A clinical investigator who enrolls patients into a placebo-controlled RCT therefore faces a conflict between his duty, *qua *scientist, to follow the experimental protocol and his duty, *qua *physician, to provide optimal care for his patients. Even when the RCT does not include a placebo control group, random assignment to a particular treatment group raises ethical concerns, because subjects may have reasons to prefer one treatment to another [20]. For example, in an RCT comparing lumpectomy to mastectomy for breast cancer treatment, many women may prefer the lumpectomy because it less disfiguring than a mastectomy [44].

Fried proposed the concept of clinical equipoise as a way of dealing with some of the dilemmas posed by RCTs [25]. An investigator can enroll a research subject in an RCT only if the different treatment groups in the RCT are in equipoise, i.e. there is genuine uncertainty about the relative merits of different treatments. When there is a therapy that has been proven to be effective, patients/subjects may receive an effective therapy or an experimental therapy thought to be effective [15]. Subjects can receive a placebo only if it is not known whether there is any therapy that is better than a placebo. If there is a therapy that has been proven to be effective, then a clinical investigator cannot enroll a patient/subject in an RCT with a placebo control group, because the investigator would be violating his duty to provide optimal medical care to the patient/subject [15].

Fried also articulated a neo-Kantian ethical justification for the doctrine of equipoise. According to Fried, patients/subjects should not be treated as mere instruments to achieve scientific or other goals [3,15,25]. Since the time of Hippocrates physicians and other health care professionals have subscribed to an ethic that requires one to protect patients from harm and promote their interests by providing optimal care and undivided loyalty [45]. This therapeutic orientation is central to medicine and other health professions because patients are vulnerable and clinicians have the expertise, knowledge, and skill to provide the protection and assistance patients require. When clinicians become investigators, clinicians do not relinquish their obligations to provide optimal medical care to their patients. Patients who become research subjects still retain their right to receive optimal care and undivided loyalty. When the role of investigator conflicts with the role of clinician, investigators should honor their roles as clinicians. Scientific considerations should not be allowed to take precedence over the good of the patient/subject.

Following Fried, other writers restated and refined the notion of equipoise [2-4,46-48]. The Helsinki Declaration codified the placebo orthodoxy that emerged from the equipoise debate. The 1989 version of the document implied that placebos should not be used in RCTs if there is already a proven effective therapy: "In any medical study, every patient--including those of a control group, if any--should be assured of the best proven diagnostic and therapeutic method" [49]. This language was later modified to allow for the use of placebos when there is a proven effective therapy and patients will not be seriously or irreversibly harmed if they do not receive it [41].

During the 1990s, commentators continued to explore the ethical issues in RCTs without seriously questioning or explicitly justifying the fiduciary approach to the investigator-subject relationship or the doctrine of equipoise. Lurie and Wolfe, for example, published an article in the *New England Journal of Medicine *in which they argued that placebo-controlled RCTs to reduce the perinatal (mother-child) transmission of the human immunodeficiency virus (HIV) being conducted in developing nations were unethical, because they violated the Council of International Organizations of Medical Sciences (CIOMS) guidelines, since a treatment had been proven effective for preventing perinatal transmission of HIV [50,51]. Though Lurie and Wolfe's article generated considerable press coverage, it lacked philosophical depth, because it did not offer a clear justification for following the CIOMS guidelines. However, the article initiated a great deal of scholarly discussion, and by the end of the decade, many writers began to take a closer look at the doctrine of equipoise and the fiduciary view.

## Critiques of the fiduciary approach

### Informed consent

The first critique of the fiduciary approach was anticipated by Hellman and Hellman as a potential objection to their analysis of the ethics of RCTs [3]. According to this critique, investigators do not need to provide patients/subjects with optimal care, provided that patients/subjects consent to receive the care available under the study protocol and understand that they are participating in research. Clinical research need not compromise the patient's rights because the patient can waive some rights when consenting research participation. A violation of a right does not occur when one waives a right [52]. Informed consent permits scientific considerations to take precedence over promoting the interests of the patient/subject [43].

One of the triumphs of the patient's rights movement is the ethical and legal right to choose treatment [29,53,54]. In the early 1970s, legal decisions undercut paternalistic medical traditions as competent patients won the right to select different treatment options, including the option of no treatment at all. The patient's right to choose also applies to the research context. During the informed consent process patients/subjects are explicitly told that they are being invited to participate in research, and they are informed about the alternatives to participating in research, such as receiving standard medical care [43]. Patients/subjects are informed about the risks of the study as well as potential benefits (if any), the purpose of the study, the length of the study, and so on [43].

Even though patients/subjects can agree not to receive optimal care, this does not mean that they may thereby be placed unduly at risk. Various laws and regulations protect research subjects from excessive risks [55,56]. All clinical trials also have inclusion and exclusion criteria, which are designed to protect patients from harm and meet the scientific objectives of the trial. Once a patient/subject enrolls in a trial, he or she will receive careful monitoring. He/she may be withdrawn from a trial to protect him or her from harm. After the trial is completed, investigators will continue to follow patients/subjects to monitor their health and protect them from harm [57].

One of the weaknesses of this critique of the fiduciary view is that informed consent in research is often far from perfect. If patients/subjects do not provide adequate consent to research participation, then they cannot waive their right to receive optimal care. A growing body of literature has demonstrated many different problems with the consent process in clinical research. Subjects often have difficulty understanding risks and benefits, the protocol design, the purpose of the research, and other crucial pieces of information [58]. Patients also often have difficulty making intelligent choices due to the effects of illness, psychological or emotional problems, socioeconomic circumstances, linguistic or cultural barriers, and so on [59]. Investigators often do not take enough time to explain their to the research and often view informed consent as a piece of paper that needs to be signed, rather than a process of communication [52].

The therapeutic misconception, i.e. the mistaken belief that clinical research studies are designed solely to promote the patient's welfare, can also undermine the consent process. The therapeutic misconception can affect many different aspects of the subject's thought processes. Patients/subjects who are under the influence of the therapeutic misconception tend to overestimate the potential benefits of research and underestimate risks. The therapeutic misconception is difficult to dispel, even when patients/subjects receive ample information about research and are well-educated, because many patients enter clinical studies hoping to find a cure for their disease [43,60].

Although there are problems with informed consent in clinical research, they do not undermine this critique of the fiduciary view, because consent often is valid and patients can often legitimately waive their right to receive optimal care. However, these problems underscore the importance of taking appropriate steps to obtain valid consent. Investigators must make sure that patients (or their representatives) have adequate decision-making capacity, receive the information they need to make a decision, understand the information, and are not facing any conditions (such as coercion or duress) that could interfere with their ability to make a free choice [55]. Investigators should also understand the social, psychological, and economic factors that can compromise consent and take appropriate measures to promote effective decision-making.

### The difference thesis

The most prolific critic of the fiduciary view has been Miller, who has published numerous articles on the subject with various coauthors [6,7,11,16,17,61]. Miller's chief criticism of the fiduciary view, known as the difference thesis, holds that the ethics of clinical research is different from the ethics of clinical practice. Clinical investigators are not bound by the same ethical obligations as clinicians who are not investigators [6]. In particular, clinical investigators do not have a duty to provide patients/subjects with optimal care.

The *Belmont Report's *distinction between research and practice, discussed earlier, serves as a key premise in the argument for the difference thesis. Clinical research and clinical practice have different ethical standards because they have different goals and methods. Clinical research is scientific in orientation, while clinical practice is therapeutic in orientation. In clinical research, tests and treatments must be administered according to a standardized protocol, so that variables can be controlled and generalizations can be drawn from the data. In clinical practice, the physician does not need to follow a rigid protocol and can offer individualized medical care to fit the patient's particular medical needs. In clinical research, a patient/subject may undergo tests and procedures designed to collect useful information, not to benefit him or her. In clinical practice, the patient should only undergo tests and procedures needed for diagnosis or treatment. In clinical research, the patient's treatment plan may be selected at random from two or more options. In clinical practice, the patient and physician choose a treatment plan that meets the patient's needs.

The difference thesis has generated considerable discussion and debate. Paul Miller and Charles Weijer challenge Miller's attempt to draw inferences about the ethics of clinical research from an account of the goals of clinical research on the grounds that the connection between goals of a practice and its ethical norms is indeterminate [48,61]. It is an empirical question which approach to the investigator-subject relationship (fiduciary or non-fiduciary) is better at developing scientific knowledge. Though there is some evidence that the non-fiduciary approach helps to advance the goal of clinical research, it may also produce some adverse effects that detract from the pursuit of those goals, such as distrust of clinical researchers. In any case, Miller needs to produce more evidence that connects the goal of clinical research to the ethics of clinical research [62].

Another objection to the difference thesis is that it is too radical: though there may be some ethical differences between clinical research and clinical practice, claiming that these activities have totally different standards is an exaggeration that threatens the welfare of patients/subjects and the public's support of the research enterprise [13,15,61]. Clinical researchers are still bound by many of the same ethical duties that apply to clinical practice, such as protecting patients/subjects from harm, maintaining confidentiality, and even beneficence. One reason why the ethical standards of clinical research may not differ radically from those of clinical practice is that clinical research may have other goals besides the development of scientific knowledge, such as caring for patients, improving public health, and so on [61]. The ethical standards of clinical research should also support these other goals, not just the goal of pursuing scientific knowledge.

Putting these two objections together, one can conclude that the most radical versions of the difference thesis are indefensible, given our understanding of the goals of clinical research and their relationships to ethical standards. However, more modest versions of the thesis are plausible. One could argue that the there are important differences between the ethics of clinical practice and the ethics of clinical research, even though there are also some similarities. These differences are sufficient to call into question the fiduciary view even if they do not justify abandoning clinical obligations to patients/subjects beyond those outlined in the protocol or those needed to protect patients/subjects from harm.

### Social goals vs. the patient's interests

The third challenge to the fiduciary view claims that ethics does not demand that the physician always provide the patient with optimal care because it is sometimes acceptable to sacrifice the patient's interests to achieve important social goals. For example, in medical education and training, patients sometimes receive suboptimal care so that medical and nursing students, interns, and others can learn, thus benefitting future patients. When a supervising physician asks a medical student to perform a rectal examination, the patient's interests may be partially sacrificed for the sake of other people, because the student is not as experienced as the supervising physician [15]. Although medical training usually does not place patients at significant risk of harm, they may be inconvenienced in order to help students learn.

Triage during disasters is another example of sacrificing the patient's interests to achieve important social goals. When there are not enough medical resources to help everyone, people who need medical aid urgently may be treated before those who do not need aid urgently and those who are so severely injured that medical aid is futile. Triage runs counter to the Hippocratic tradition of putting the patient first, since it requires health care professionals to consider how to best help the most patients [63].

A potential problem with the social goals challenge to the fiduciary view is that it embodies a utilitarian rationale that could be used to justify unethical practices, such as conducting studies on people without their consent, exposing people to excessive risks, exploiting vulnerable subjects, and so on [35,36]. To avoid these undesirable implications, it is important to set some limits on the sacrifices that patients/subjects may be required to make for medical research. For example, risks must be minimized, informed consent must be obtained, vulnerable subjects must be protected, and so on [56]. Although the social goals critique must be properly qualified, it still presents a significant challenge to the fiduciary view. It is reasonable to suppose that the social goals promoted by research may sometimes take precedence over the interests of the patient/subject, provided that appropriate protections are in place.

### Alternatives to the fiduciary approach

Each of these critiques reveal significant flaws with the fiduciary view, and, taken together, they show that the fiduciary view, is untenable.^c ^Investigators do not always need to provide patients/subjects with optimal care, and they may sometimes allow scientific considerations to take precedence over the interests of the patient/subject, provided that other safeguards are maintained, such as informed consent and risk minimization. If the fiduciary approach of the investigator-subject relationship is flawed, what should take its place? I will now discuss two alternatives.

### The non-exploitation approach

Miller and colleagues have developed an account of the investigator-subject relationship dubbed the non-exploitation view. The main argument for this position is the difference thesis, discussed above. Because the goal of research is to develop knowledge, not to benefit the patient, clinical investigators are not required to provide subjects with optimal care. Their main ethical obligation is to avoid exploiting human subjects [6,7]. Non-exploitation should be a guiding principle in research ethics because most of the existing laws and guidelines were developed, in part, to prevent exploitation of human subjects in research and the worst abuses of human subjects have involved exploitation [16]. One implication of this approach is that the doctrine of equipoise is incoherent and should be abandoned, because equipoise is founded on the notion that investigators have a duty to provide optimal care to the patient [16]. Placebo-controlled RCTs should be allowed when they are supported by sound methodology and do not expose human subjects to excessive risks [6,7].

There are two significant problems with the non-exploitation view, however. The first is that Miller and colleagues have not provided an adequate account of exploitation in biomedical research. In one of their early discussions of the non-exploitation view, Miller and Brody said that patients/subjects are not exploited if they are not exposed to excessive risks and if they understand that they are participating in research and not receiving "personalized care directed at their best interest" [[6] at p. 5]. Miller and Brody later added that seven ethical principles of clinical research ethics defended by Emanuel and colleagues [56] provide a framework for protecting research subjects from exploitation [7]. Recently, Miller and Brody acknowledged that their account of exploitation is underdeveloped [16].

If the exploitation were straightforward and uncontroversial, then the failure to fully articulate a notion of exploitation would not be a major weakness of the non-exploitation view. However, exploitation is complex and controversial. There are a variety of different accounts of exploitation in the literature, ranging from Marxist theories to free-market approaches [64]. Moreover, the relationship between providing medical benefits and avoiding exploitation in clinical research is hotly contested [65]. Some authors argue that to avoid exploitation in developing countries investigators must provide medical benefits to the subjects and address underlying injustices in the host community or country, while others argue that investigators can avoid exploitation by providing subjects and the community or country with a fair share of the benefits of research [66,67]^d^.

The second problem with the non-exploitation approach is that proponents of this view have not clearly stated how much clinical care investigators owe patients/subjects. At times, the authors suggest that investigators have no duties to provide patients/subjects with clinical care beyond what is required to achieve the scientific aims of the study. For example, Miller and Brody state that "clinical research...is not a therapeutic activity devoted to the personal care of patient" [[7] at p. 21]. But Miller and Rosenstein also observe that "physician-investigators have a responsibility to provide appropriate medical attention and care at the same time that they engage in scientific investigation" [[68] at p. 1385] and Miller and Brody admit that "a physician-investigator has an obligation to maximize therapeutic benefits to the patient-subject, provided that this benefit can be attained within the scientific constraints imposed by the protocol" [[16] at p. 163] So how much clinical care do investigators owe patients/subjects? It is difficult to pin Miller and colleagues down on this issue [61]. All that can be said with confidence is that they believe that investigators do not need to provide patients/subjects with optimal care.

It is important to have a clear understanding of how much clinical care investigators owe to patients/subjects, since this is the critical issue in the debates about RCTs, placebo-controls, drug washout periods, and other issues related to the investigator-subject relationship. All commentators on these topics agree that clinical investigators must provide patients/subjects with the clinical care described in the protocol as well as the care needed to protect patients/subjects from harm. But what should they do for patients/subjects beyond this minimal standard? That is the crux is the matter.

### The partial entrustment approach

Richardson and Belsky develop an alternative to the fiduciary view that attempts to deal with the question concerning the extent of the clinical investigator's obligation to provide the patient/subject with clinical care. The authors reject the extreme view that investigators have no duty to provide patients/subjects with medical benefits beyond what is required by the protocol, arguing that there are two sources of justification for investigators' therapeutic obligations. First, all moral agents have a duty of rescue, i.e. the obligation to make a reasonable effort to help a person who is endangered[9]. The duty of rescue is different from a duty to avoid causing harm to other people, since it is a positive duty to aid someone. Various moral theories, including Kantianism, utilitarianism, and social contract theory, support a duty to help others, which implies a duty of rescue [53].

Second, investigators have special moral obligations beyond the duty of rescue as a result of their relationships to patients/subjects. Because investigators have considerable discretion to make decisions that affect the welfare of patients/subjects and because patients/subjects are vulnerable, investigators are partially entrusted to promote the welfare of patients/subjects. Richardson and Belsky call their view a "partial entrustment" approach to distinguish it from the fiduciary view, which involves complete entrustment [9]. Richardson and Belsky argue that the relationship between the investigator and patient/subject is similar to a legal relationship known as a bailment. In a bailment, the entrusted person, the bailee, agrees to take care of an item owned by another person, the bailor, and return it undamaged. For example, in valet parking, the company has a responsibility prevent the car from being damaged while under its care and to return the car to the owner in good condition [69].

Richardson and Belsky describe some factors that determine the extent of the investigator's clinical responsibilities to human subjects, including the nature of the research, the investigator's resources, the subjects' vulnerabilities, and what the subjects have consented to. For example, an investigator who is conducting research on highly vulnerable subjects, such as patients with cognitive impairments, would have greater responsibilities to the subjects than an investigator who is conducting research on subjects who are not as vulnerable, such as healthy volunteers donating biological materials to a biobank. An investigator who is conducting a Phase III oncology clinical trial would have greater responsibilities than an investigator who is collecting blood and tissue for a biobank, because the oncology investigator has more discretion than the biobank investigator [9].

Richardson and Belsky apply their analysis to a case involving ancillary care, i.e. care that a clinical investigator may offer to a research subject that is not required by the scientific aims of the study. The authors argue that investigators who are studying malaria in children living in an area of Africa where the disease is endemic have a responsibility to diagnose and treat schistosomiasis, a parasitic disease caused by flatworms, which is likely to be found in up to 10 percent of subjects. Diagnosing schistosomiasis is within the investigator's discretion because the disease is easily detectable in urine samples drawn for the study and up to 10 percent of children are likely to have the disease. Additional factors in favor of diagnosing and treating the patients/subjects include their high degree of vulnerability and the investigators' ample resources. However, if the prevalence of the disease were 90 percent, this would greatly weaken the obligations to treat the disease, because this would put an enormous burden on the research team's budget, which could make it difficult to conduct the study, according to the authors [9].

Richardson and Belsky's analysis of the investigator-subject relationship is insightful, sophisticated, and well-reasoned. In my judgment, it is the best model of the investigator-subject relationship proposed thus far. It embraces the widely accepted idea that investigators have some responsibilities to provide clinical care to patients/subjects beyond what is required by the protocol or is necessary to protect patients/subjects from harm, but it also specifies conditions for delineating those responsibilities. However, the partial entrustment model has some flaws as well.

First, the notion of a partial entrustment is morally unstable, because it may easily degenerate into no entrustment if the party who is partially entrusted attempts to limit entrustment via legal maneuvers. For example, businesses often try to avoid legal responsibilities by posting warnings, such as "not responsible for lost or stolen goods," "no lifeguard on duty; swim at your own risk," etc. People who read signs like these are likely to wonder whether the business can be trusted at all. Likewise, investigators may attempt to limit entrustment through consent process, which would tend to make patients/subjects wonder how much the investigator can be trusted. Complete entrustments cannot be easily disavowed, but partial entrustments can be. The notion of "partial entrustment," like the notion of a "half-truth," invites skepticism and suspicion, and is not a sound foundation for investigator-subject relationship.

Second, the legal concept of bailment cited by the authors as an example of a partial entrustment typically does not involve significant beneficence obligations on the part of the bailee toward the bailor [69]. A valet parking company has an obligation to protect the vehicle from damage, but it need not take any steps to improve the vehicle, such as changing the oil or replacing the muffler, unless is has specifically agreed to do so. This is very different from the example of ancillary care mentioned by Richardson and Belsky. A researcher who provides ancillary care is like the valet parking company that also changes the oil and replaces mufflers.

Third, there are factors that affect the investigator's obligations to the subject that Richardson and Belsky do not consider in adequate depth, such as the investigator's expertise. On the one hand, an investigator cannot be expected to provide a benefit that he/she is not qualified to deliver, no how matter vulnerable the patient is or how many funds the investigator has available. For example, a neuroscientist who sees an image that appears to be a tumor on an MRI scan is not qualified to make a diagnosis, though a radiologist would be. On the other hand, if an investigator has sufficient expertise to provide a benefit, then he/she may be obligated to provide it. For example, a radiologist who reads an MRI scan as part of a research study would have a duty to determine whether an image on the scan is likely to be a tumor.

In sum, while these two alternatives to the fiduciary view have redeeming features, they also have some flaws. There is a need, therefore, to develop a better alternative to the fiduciary view, which will be my main concern in the remainder of this essay.

### The contextual approach

The approach I will present builds on Richardson and Belsky's key insight that the investigator's obligation to provide clinical care can vary from one situation to the next. Many commentators have tried to resolve the conflict between the investigator's clinical and scientific roles by siding with one role or the other. The fiduciary view tips the scales in favor of the clinical role over the scientific one, while the non-exploitation view tips the scales in the other direction. Though these two polar views are fairly simple and easy to understand, the correct view, I shall argue, is more nuanced than either of these. The balance can shift from one role to the other, depending on the context. In some situations, the role of the clinician should dominate; in others, the role of investigator should dominate. And there are many variations in between.

To generate some intuitive support for a contextual approach, consider the wide variety of activities that are classified as clinical research. Clinical research includes: Phase I, II, III, and IV clinical trials; environmental exposure studies; genetic research; epidemiological research, public health research, behavioral studies; and case reports [57,70]. If ethical obligations depend, at least in part, on factors inherent in the particular situation [53], it is reasonable to suppose that the investigator's ethical obligations to the patient/subject may be different in these different types of research. It seems plausible that the obligations of an investigator conducting a Phase I drug safety study on healthy volunteers are different from those of an investigator conducting a Phase III drug clinical trial on patients with a disease.

While the idea that obligations to the patient/subject may be different in different situations has intuitive appeal, it requires a philosophical foundation, so that we have a clearer account of the investigators' obligations to the patient/subject. To develop the conceptual underpinnings of the contextual approach, I will make a few comments about institutional (or role) morality.^e^

In society, people have general ethical obligations as well special obligations associated with particular institutions, such as business, government, medicine, science, marriage, family, and so on [71,72]. General, ethical obligations apply to all people who are capable of following moral rules (i.e. moral agents), but institutional obligations, apply only to people who occupy specific roles in those institutions. The justification for institutional morality is twofold: first, institutional ethical standards must help to promote the goals of the institution; and second, the institution itself must be morally worthwhile [73]. For example, confidentiality is ethically justified in medicine because it promotes doctor-patient trust, which is necessary for effective diagnosis and treatment; further, medicine is a worthwhile social institution [53]. Secrecy in organized crime is not justified because organized crime is an immoral activity.

Very often, people can avoid conflicts of institutional obligations through prudent decision-making and planning. When conflicts cannot be avoided, one must decide which obligation should have priority by considering the circumstances (i.e. contextual factors) and options, in light of ethical theories or principles [74]. Suppose that a man, John, is a volunteer firefighter. He also has a 12-year-old son who is studying the piano. His son has a recital at the same time the firefighters have an organizational meeting, which cannot be changed. John decides to go to the recital and skip the meeting. He knows that his son will be greatly disappointed if he misses the recital, and he is not needed at the meeting. If the circumstances were different, John would be justified in deciding to forego the recital. For example, if John is needed to help fight a fire at a hardware store just before his son's recital, John would decide to give priority to his obligations as a firefighter, since many people could be harmed if the fire is not controlled.

We can apply the insights from these simple examples to more complicated ones involving science and medicine. Very often clinical investigators can fulfill their different institutional roles without having to deal with any conflicts. When unavoidable conflicts occur between different institutional obligations, as is the often the case in RCTs, the clinical investigator must decide which obligation should have priority. To deal with conflicting institutional obligations, the investigator should consider the circumstances (or context) of the research, available options, and ethical principles or norms, such as risk minimization, informed consent, protection of vulnerable subjects, etc. The circumstances can have a profound impact on decision-making, as they can make the difference between favoring one role over another. The following contextual factors, some of which have been mentioned previously, are relevant to balancing clinical and scientific obligations:

• **The research design**. Research design can affect many contextual factors, such as benefits and risks. Key elements of the design include the methods and procedures, inclusion and exclusion criteria, and data and safety monitoring plans. Sometimes it may be possible to alter the study design to reduce risks or increase benefits without adversely affecting the likelihood of achieving the aims and objectives of the study. For example, a study might be changed from a placebo control group design to an active control group design, which could potentially increase benefits to the subjects [5,75].

• **Benefits and risks**. In general, the investigator's clinical role increases as the medical or psychological risks to the subject increase, because the investigator must take additional steps to protect the subject from these risks. The level of risk may also have an impact on the expertise that is required of the investigator. For example, if a study includes a bronchoscopy, an appropriately trained physician should perform this procedure. The potential social benefits of the research may also impact the investigator's clinical obligations. As noted earlier, the patient's interests can sometimes be partially sacrificed for research that is likely to achieve important social goals, provided that other protections are in place. Many writers argued, for example, that the use of placebos was justified in the clinical trials criticized by Lurie and Wolfe, because one of the main goals of the research was to develop an affordable method for preventing the perinatal transmission of HIV in developing nations, which could save thousands of lives [76].

• **The patient/subject's reasonable expectations**. The patient/subject's reasonable expectations need to be considered to maintain the patient/subject's trust, which is essential to both clinical research and clinical medicine [43,56]. Reasonable expectations are explicitly addressed during the informed consent process, but one must also consider the patient/subject's assumptions about the research. For example, if the patient is desperately ill and views research participation as her best hope, she would probably regard research as potentially therapeutic [55]. Although investigators should not perpetuate the therapeutic misconception, they need to be aware of the patient/subject's expectations concerning potential medical benefits.

• **The patient/subject's vulnerability**. The patient/subject's vulnerability can affect his or her ability to consent to research participation and to promote his or her own interests [59]. People who are highly vulnerable may have greater medical, psychological, and economic needs than people who are not as vulnerable and require additional protection from risks related to research participation [9,77]. One could also argue that principles of justice demand that investigators give special attention to protecting and caring for those most in need of help [42,78].

• **The patients/subjects motivations**. Some commentators have argued that the patient/subject's motivations can make a difference in the ethics of research. For example, someone who enrolls in a study for altruistic motives may be more willing to forego medical treatment than someone who enrolls in a study for personal benefit [79]. While motivations can have an important bearing on the investigator-subject relationship, they can be very difficult to assess. People may espouse altruistic motives but still be interested in receiving medical benefits.

• **The investigator's ability to benefit the subject**. This factor has a significant impact on the investigator's obligations to provide medical care for the patient/subject, since it is not reasonable to require someone to do something they are not capable of doing [80]. The investigator's ability to benefit the subject is a function of several factors, including his or her **resources **(discussed earlier), **expertise **(also mentioned earlier) and **knowledge**. Knowledge needs to considered because there is no a duty to render aid to a person if one does not know about that person's needs or would not be reasonably expected to know about them.

• **The investigator's previous relationship to the patient/subject**. Though patients are often referred to research studies by their primary care physicians, sometimes investigators have prior relationships with the potential research subjects they are recruiting. An investigator who enrolls one of his or her own patients into a study should not be able to easily shed obligations to benefit the patient, due to their prior relationship. It may also be especially difficult for the patient to grasp the idea that the purpose of the study is to obtain generalizable knowledge, not to benefit him or her, when the patient already has a therapeutic relationship with the investigator [9]. Under these circumstances, investigators must be especially wary of the therapeutic misconception and the patient's expectations to receive medical care [60].

**Figure 1:**
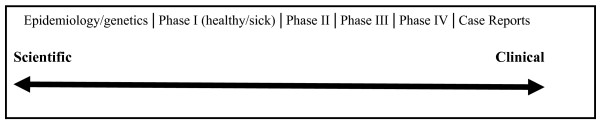
**Balancing Scientific and Clinical Roles**.

These seven contextual factors--and possibly more--play an important role in deciding how to settle conflicts between an investigator's scientific and clinical obligations. I will now illustrate this point by examining different types of clinical research (see Figure 1).

## Applications

### Epidemiologic research

Epidemiologic studies involve the observation of human subjects in their natural environment, without the provision of medical treatment or any other controlled intervention. Some common epidemiologic research methods include prospective cohort studies, retrospective case-control studies, and cross-sectional studies. Epidemiologists may collect biological samples, and health and demographic information. The purpose of epidemiologic research is not to test specific treatments, but to develop general knowledge about the relationships between diseases and causal factors, such as diet, lifestyle, environmental exposures, and genetics [57].

A strong argument can be made that the investigator's main role in epidemiologic research is scientific, not therapeutic. First, epidemiologic studies usually involve minimal risks. In many studies, the biggest risk of research participation will be potential loss of confidentiality or bruising or infection from a blood draw [57]. Second, since epidemiologic studies involve the collection of data but not medical treatment, patients/subjects are not likely to expect that they will receive medical care. Third, the investigator's ability to help the patient/subject may be quite limited, due to his or her lack of expertise, knowledge, or resources. He or she may also not have access to any data that could be useful in diagnosis or treatment. The investigator may not even have access to information that identifies the patient/subject, since many epidemiologic studies anonymize the data [57]. Fourth, the investigator probably has no prior relationship with the patient/subject. Epidemiologic studies are often conducted by people who specialize in a particular discipline and are not practicing medicine on the patients they are studying.

Although investigators have minimal obligations to provide clinical care in most epidemiologic studies, there are some exceptions to this rule. Suppose an epidemiologist is also a physician who is studying a population in a developing nation and that he has been providing medical care to many of the people in the community. In these circumstances, one could argue that he has some obligations to provide medical benefits to patients/subjects, provided that he has the resources to do so, because they are vulnerable, he has the requisite expertise, and he has a prior relationship with them. For example, if he is conducting medical examinations or tests as part of his study, he could make recommendations for medical treatment if he discovers problems that warrant it, such as infectious diseases.

### Genetic research

Many types of clinical research studies examine the relationship between genes and diseases. Studies may attempt to explore statistical relationships between patterns of genetic variation and diseases, identify or sequence specific genes that increase the risk of developing diseases, or investigate the causal pathways from genes to disease. In these studies, investigators collect biological samples (such as blood, buccal cells, or hair) from human subjects and obtain information about their medical history, lifestyle, and environmental exposures. The goal of the genetic research is usually not to develop new medical therapies but to gain a better understanding of the genetic basis of health and disease, which may lead to new therapies [57].

Like epidemiologic research, the investigator's main role in genetic research is usually scientific, not therapeutic. First, genetic research, like epidemiologic research, usually involves minimal risks. Second, since patients/subjects are not receiving therapy as part of the study design, they are not likely to expect to receive therapy. Third, the investigator's ability to help patients/subjects may be quite limited, due to lack of expertise, knowledge, or resources. The investigator may be a geneticist with little or no background in the area clinical medicine that is relevant to patients/subjects' needs, and the investigator may not have access to clinically useful information about patients/subjects. Like the epidemiologist, the geneticist may not even have access to data that personally identifies patients/subjects. Fourth, the genetic researcher, like the epidemiologist, probably has no prior relationship with the subjects.

Although researchers have minimal clinical duties in genetic research, there are also some exceptions to this rule. If an investigator discovers some clinically useful information about a research subject, such as a gene strongly associated with a type of cancer, an argument can be made that the investigator should share this information with the subject and make an appropriate referral, because the subject could benefit from receiving this information, and it would not take too much effort to provide it [19,80]. However, the decision to share genetic information with patients/subjects creates its own dilemmas, since the genetic tests may not be well-validated and the meaning of the genetic information may not be well-understood. Some investigators inform patients/subjects up front that they will not provide them with the results of any genetic tests, so they can avoid the dilemma of whether (and how) to inform patients about these results [81].

Investigators who combine genetic studies with other types of research, such as clinical trials, may also have extensive clinical obligations. For example, in pharmacogenetics research investigators attempt to understand how genetic variations affect drug absorption, transport, removal, metabolism, etc [82]. An investigator who is conducting a Phase III clinical trial that aims to determine whether there are genetic factors related to how patients/subjects respond to a diabetes medication would have clinical responsibilities similar to another investigator conducting a Phase III study (for more on clinical trials, see below).

### Phase I trials

Phase I is the first stage in human testing for new treatments, such as drugs, biologics, and medical devices. Phase I trials are designed to obtain data pertaining to safety, not efficacy. They are usually conducted on healthy volunteers, though some Phase I studies are conducted on patients with advanced cancer or other diseases, who may be able to benefit from receiving the treatment. Phase I studies are usually small (20-100 subjects) and have a short duration (a few months). Phase I studies are usually conducted in a clinical setting, such as medical center or hospital, so that investigators will have the resources necessary to monitor research subjects and provide emergency medical care, if necessary. Subjects are usually paid a considerable sum of money for their participation [83].

A strong case can be made that the clinical investigator's primary role in a Phase I trial in healthy volunteers is scientific, not therapeutic, even though he or she has more clinical responsibilities than someone conducting epidemiologic research. First, healthy volunteers probably do not expect to receive any medical benefits. They are likely to view their participation as a way to earn some money and help advance scientific research, not as a means of obtaining therapy [84]. Second, though the subjects may be somewhat vulnerable, due to their socioeconomic circumstances, they are probably not highly vulnerable. Phase I studies on healthy volunteers are usually conducted on adults without significant cognitive impairments [83]. Third, the clinical investigators usually have no prior therapeutic relationships with the subjects. Clinical investigators conducting Phase I studies on healthy volunteers are, for the most part, researchers studying the adverse effects of treatments in people, not clinicians who are helping to advance medical science. Fourth, since Phase I studies often involve significant risks to human subjects, investigators do have clinical responsibilities related to protecting subjects from harm, such as careful monitoring, provision of emergency medical treatment, and so on. Investigators in Phase I studies must also have the appropriate expertise to conduct studies that induce toxicity in patients/subjects.

The circumstances are much different when a Phase I trial involves patients with diseases. First, although Phase I studies on patients are designed to test safety, not efficacy, there is often a small chance (5% or less) that patients may benefit from their participation, and it may sometimes be possible to alter the study design to maximize potential benefits[85]. Second, diseased Phase I patients/subjects may expect to receive medical benefits from their participation. Research shows that patients in Phase I oncology trials often expect to benefit from their participation even when they are explicitly told that they are not likely to [85]. Third, subjects are usually highly vulnerable, due to their illness. Subjects in these trials are usually very sick and view participation in research as their last hope [85]. Fourth, the clinical investigators may have a prior relationship with the patients/subjects. For example, an investigator may be an oncologist who has been treating a patient, who suggests that the patient consider participating in a clinical trial when the present treatment is no longer beneficial. Fifth, investigators probably have the knowledge and expertise necessary to provide the patients with medical benefits beyond what is required by the study, though their resources may be limited.

### Phase II & III trials

After a new treatment has completed Phase I testing, Phase II testing may begin. During this stage, the drug is administered to patients with a disease that the drug is intended to treat, and data are collected regarding the drug's efficacy and safety. Phase II studies typically include 100-300 patients and may last up to two years. Patients are randomly assigned to receive the new drug, an effective therapy, or a placebo, depending on the study design. Investigators take various steps to minimize risks to subjects, such as excluding people who may be too ill to benefit from the drug and periodically reviewing participants' clinical data. A Phase II trial may be stopped if a drug is too dangerous or ineffective. If a drug has proven to be safe and effective in Phase II testing, a larger, Phase III study may be initiated. Phase III studies may include many thousands of patients and last for many years [70]. Subjects in Phase II and III studies are usually provided with treatment at no cost and they may or may not be paid. A product that successfully completes Phase III testing may be submitted for regulatory approval [57].

One could argue that the investigator's obligations to provide patients/subjects with optimal care are stronger in Phase II and III trials than in Phase I trials. First, the patients/subjects in Phase II or III are more likely to view their participation as a form of therapy than patients/subjects in Phase I, because they may be receiving some form of treatment as part of their participation. Second, the patients/subjects may be vulnerable in Phase II and III studies, due to their illness. Third, investigators usually have the expertise and knowledge to offer patients/subjects clinical care beyond what is required by the protocol. Fourth, investigators may have prior relationships with the patients/subjects. For example, an investigator might be an infectious disease specialist who presents his patients with an opportunity to enroll in a research study that might benefit them. They often enroll patients in research studies while simultaneously providing routine care.

As mentioned previously, using placebo control groups presents a difficult challenge for balancing clinical and scientific roles in Phase II and III studies. It is important to note that virtually all commentators agree on two points. First, placebo controls are acceptable when there is no effective treatment for the disease under investigation, because this would not deny patients any medical benefits. Second, placebos should never be given to a human subject when this is likely to cause serious or irreversible harm, since investigators have a duty to minimize risks to subjects. For example, it would be unethical to give a placebo to someone with diabetes who is currently taking insulin to control his blood sugar levels, as the subject could become severely ill and possibly die if he stops taking his medication. Likewise, some have argued it would be unethical to give placebos to patients who are taking anti-psychotic drugs to control their condition, as this could cause a relapse of their illness and lead to suicide or homicide [86].

The most controversial questions concerning placebos involve situations in which there is an effective treatment, but giving patients/subjects a placebo will not cause serious or irreversible harm, because they are not currently taking the treatment, due to their socioeconomic circumstances. The fiduciary approach holds that it would be unethical to enroll a patient in study when there is an effective treatment, since the investigator has an obligation to provide the patient with optimal care. The non-exploitation view holds that it would be ethical to enroll a patient under these conditions, provided that exploitation is avoided. According to the approach I am proposing, the ethics of the study would depend on resolving several contextual issues. First, could the study design be altered so that an active control group is used instead of a placebo, without impacting the validity or clarity of the results? There is an ongoing dispute as to whether placebo-controls are always necessary to achieve scientific aims [75]. Second, would it be reasonable for the subjects to expect to receive medical treatment? Do they understand that they may not benefit from participating in the study? Third, are the subjects vulnerable? How does their vulnerability impact their decision-making? Fourth, does the investigator have the ability to help the subjects? Are there sufficient resources to conduct a trial in which the control group receives an effective treatment? Fifth, does the investigator have a prior relationship with the subjects? Finally, what is the social value of the study? Does the value of the study justify using placebo controls? The answers to these and other questions concerning the circumstances of the research can help determine whether placebo control groups are acceptable.

One reason why the HIV trials criticized by Lurie and Wolfe were so controversial is that some of the contextual factors were disputed and others pulled in opposite directions. The issue most disputed was whether the aims of the research could have been achieved with active controls. Critics, such as Lurie and Wolfe [50], argued that placebo-controls were unnecessary, while proponents, such as Varmus and Satcher [87], countered that placebo controls were necessary. Critics and proponents of the studies agreed that the research had a socially valuable objective--to develop an affordable treatment for preventing the perinatal transmission of HIV--but they did not agree that pursuit of this objective could justify giving some subjects a placebo [76].

### Phase IV (post-marketing studies)

Phase IV trials occur after a product has been approved for distribution and marketing. During this phase, the manufacturer may gather additional data on the product's safety and efficacy on tens of thousands of people and compare it to other treatments. Information is also obtained on the long-term effects of the product and its impact on quality of life. The product may be tested in populations that were not included in Phase II and III studies. Phase IV studies often do not use some of the methods used in Phase II and III, such as control groups, randomization, or blinding [57].

One could argue that the shift from the scientific role to the therapeutic role continues in Phase IV. First, by the time a new therapy reaches Phase IV, it is no longer experimental or untested. Hence, it is more likely to offer benefits to patients/subjects. Second, since the methods used in Phase IV studies are also often less rigorous than those used in Phase II or III, the demeanor of a Phase IV may less scientific than the demeanor of a Phase II or III study. Third, the investigators are often clinicians gathering data in their clinics, not academic scientists, so they are likely to have prior relationships with their patients/subjects.

### Case reports

The final type of research I will consider is the case report. A case report involves the careful description of a case (or series of cases) that a clinician has encountered in his or her practice, such as a patient with rare disease, or unusual symptoms, findings, treatment, or pathology. A clinician who is planning to write a case report about a patient need not significantly alter his or her practice for the sake of the research, since the main goals are to diagnose and treat the patient, and research goals are secondary. Additional data or samples may be gathered for the case report, and a patient may be asked to give permission for the use of his photos or medical records, with identifying information removed. Though case reports lack the rigor of clinical trials or epidemiological studies, they can provide some useful information for researchers and clinicians. Prestigious journals, such as the *New England Journal of Medicine *and *the Journal of the American Medical Association *regularly publish case reports [57].

In case reports, the clinical role clearly dominates. First, the treatment plan is tailored to meet the patient's specific needs, not to test any scientific hypotheses. Second, patients expect to receive medical treatment and are usually not altruistically motivated. Third, patients are often vulnerable, due to illness or other factors. Fourth, the investigators usually have the knowledge, expertise, and resources to benefit the patients. Fifth, the benefits and the risks to the patient receiving treatment may be high, depending on his or her disease. Sixth, the investigator often has a prior relationship with the patient before the encounter that led to the case report.

## Conclusion

To summarize, clinical investigators face a conflict between institutional roles (clinician vs. scientist), which can generate different ethical obligations. To deal with the conflict, investigators should weigh and consider their options in light of ethical theories and principles and the contextual factors, such as the research design; benefits and risks; the patient/subject's reasonable expectations, motives, and vulnerability; their ability to help the patient/subject; and their prior relationships with the patient/subject. The investigator's obligation to provide clinical care to the patient/subject can vary, depending on the circumstances. In some situations, the clinical role dominates, and the investigator has significant obligations to provide the patient/subject with medical benefits; in other situations, the scientific role dominates, and the investigator has minimal obligations to provide the patient/subject with medical benefits. And there are many variations on these patterns.

Before concluding this essay, I will address one objection and make one comment about policy implications. First, one might object that my contextual approach is a form of moral relativism, since the investigator's ethical obligations may be different in different situations. Moral relativism in human subjects research is objectionable because it can be used to justify abuses of human subjects in the name of science and leads to inconsistency, i.e. double-standards [88]. My response to this charge is that ethics can be situational without being relativistic because there are some universal standards that apply to different situations [89]. These standards include some principles discussed in this essay, such as minimizing risks and obtaining informed consent, as well as others discussed elsewhere, such as safeguarding confidentiality and privacy, independent review of research, and equitable selection of subjects [56]. To apply an ethical principle to a particular situation, one must interpret the principle in light of the facts at hand and balance the principle against other ethical principles and considerations. This view is not an "anything goes" form of relativism because the same standards apply universally, even though they may be interpreted and implemented differently. For example, informed consent in an African village may be very different from informed consent in Boston, MA, due to social, cultural, and linguistic differences [56].

Second, earlier I stated that this essay addresses the ethical duties of investigators, not their legal duties. However, one could argue that my views have some implications for the law. For instance, one could argue that judicial opinions that examine the legal duties of clinical investigators should consider the context of research, or that interpretive guidance issued by regulatory agencies should address contextual factors. I will not pursue that line of argument here, but leave it for others to explore.

## Competing interests

The author declares that they have no competing interests.
